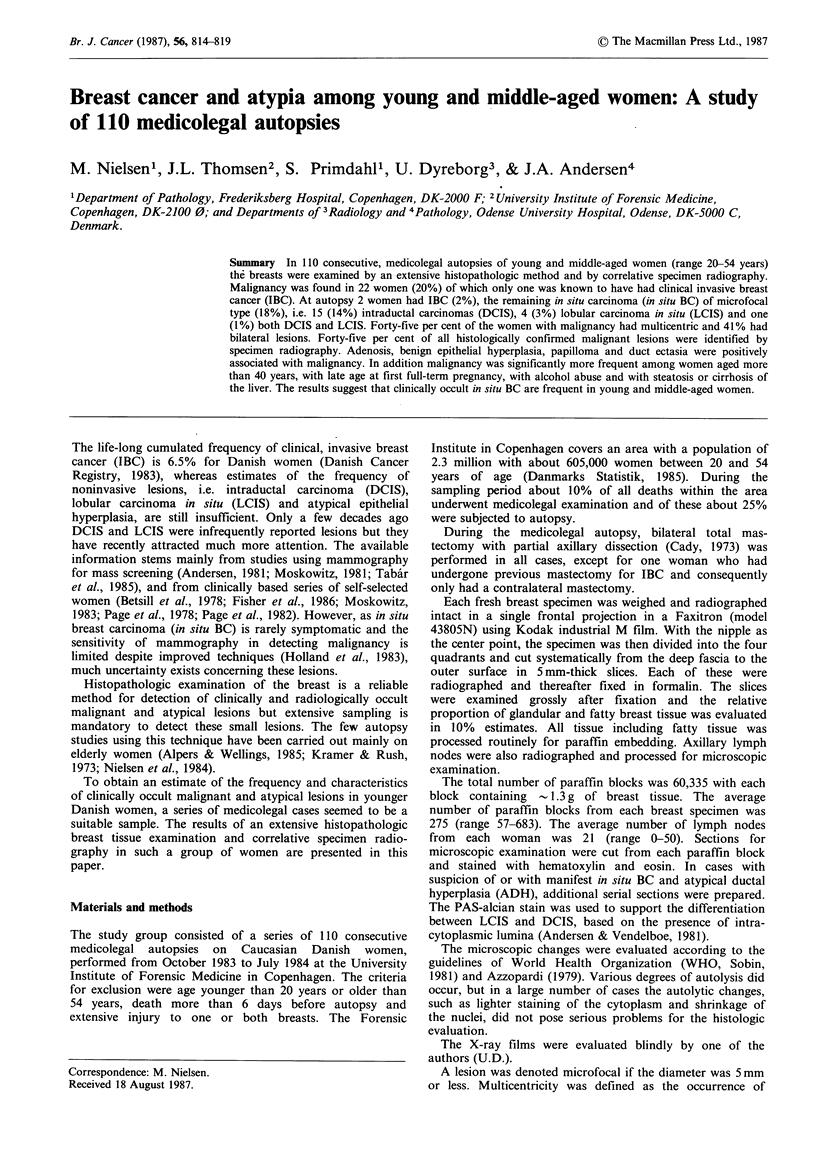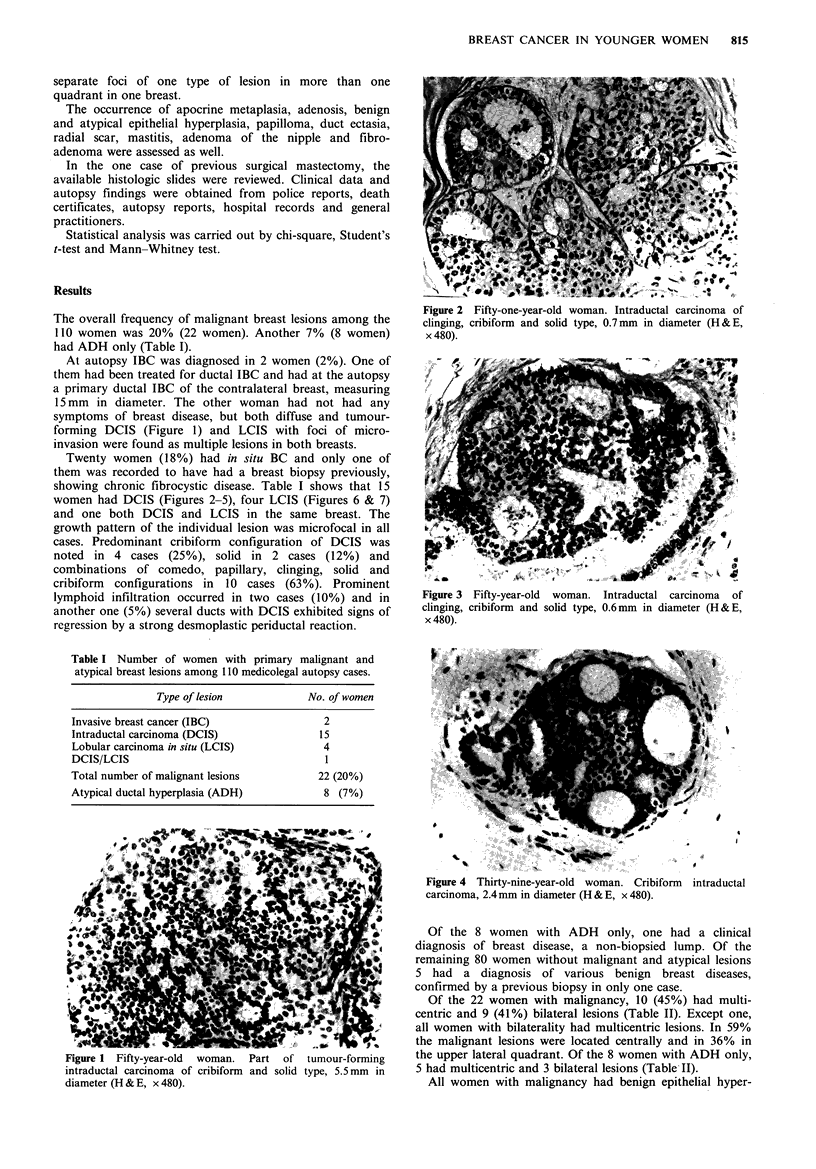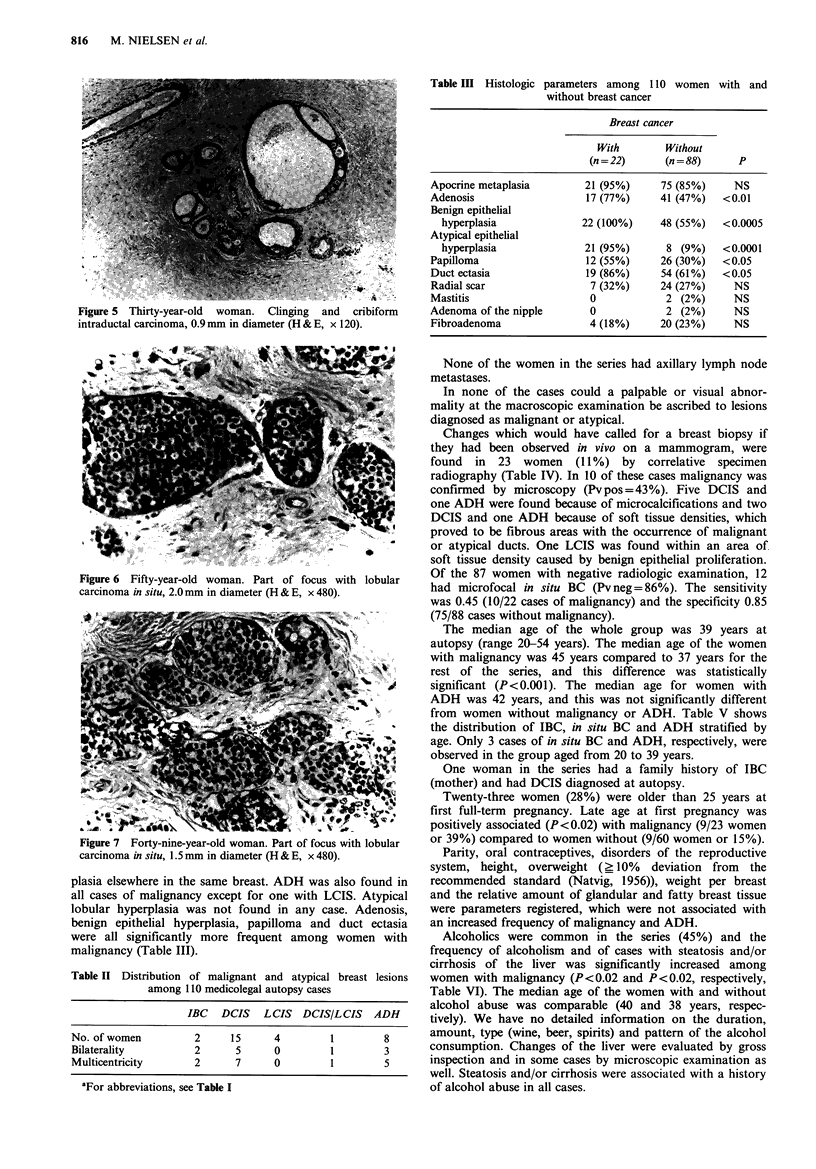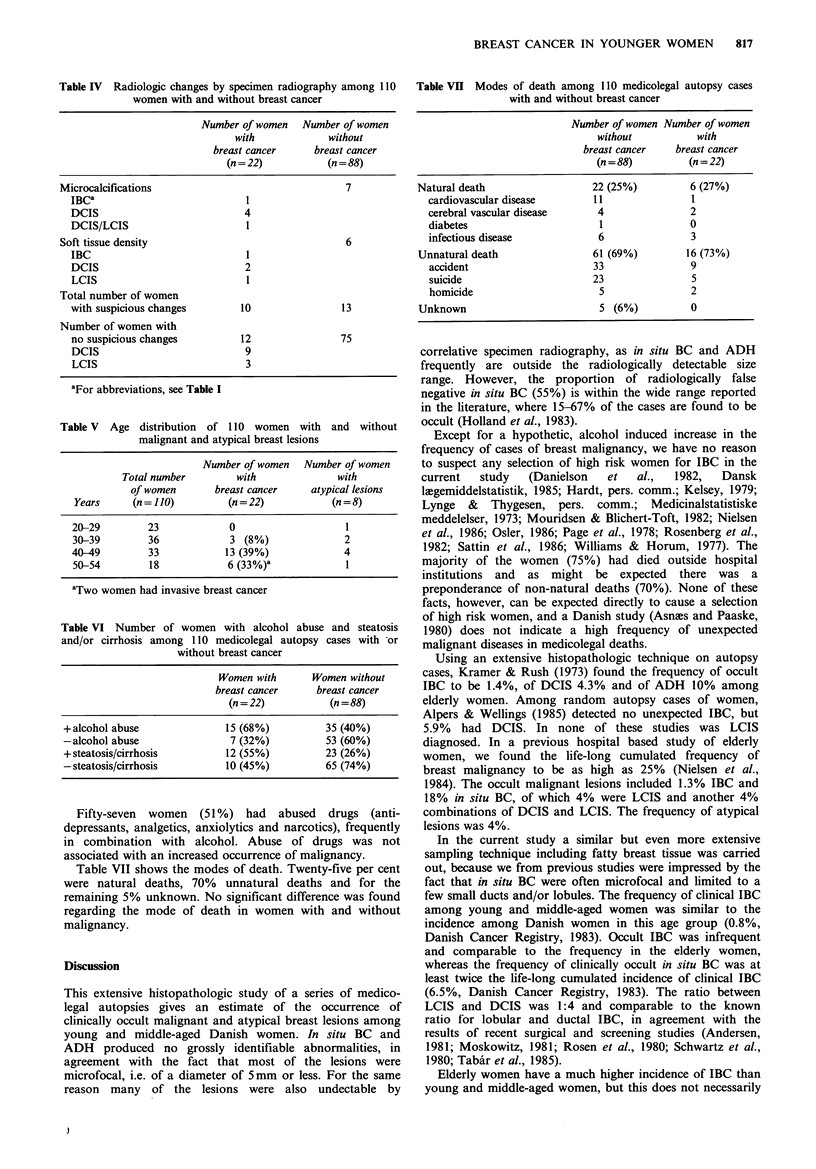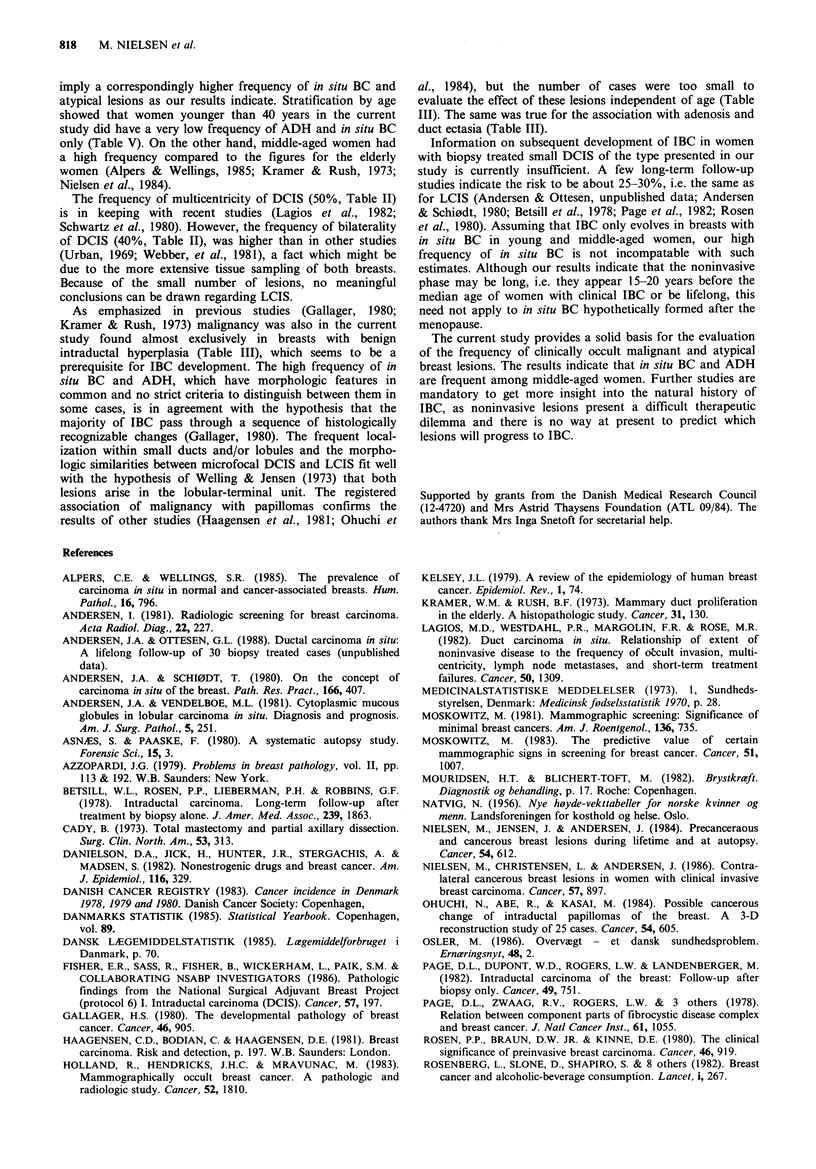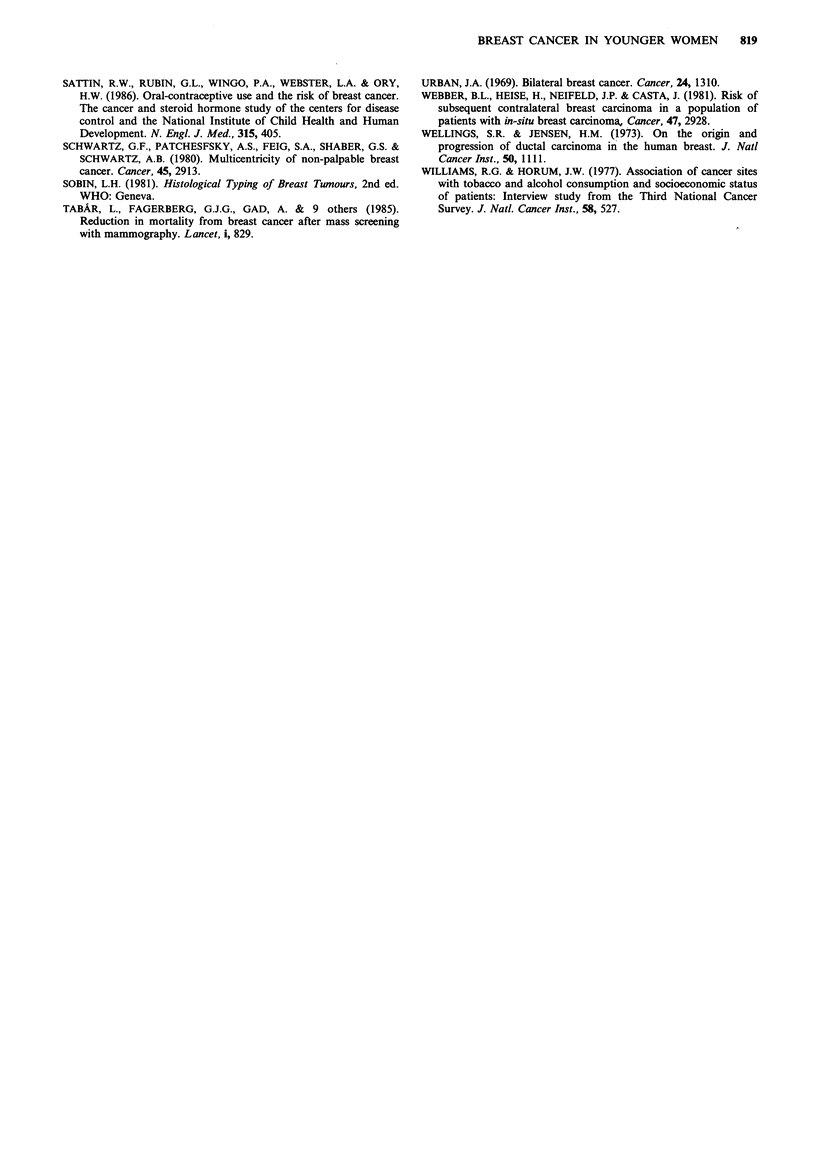# Breast cancer and atypia among young and middle-aged women: a study of 110 medicolegal autopsies.

**DOI:** 10.1038/bjc.1987.296

**Published:** 1987-12

**Authors:** M. Nielsen, J. L. Thomsen, S. Primdahl, U. Dyreborg, J. A. Andersen

**Affiliations:** Department of Pathology, Frederiksberg Hospital, Copenhagen, Denmark.

## Abstract

**Images:**


*Br. J. Cancer* (1987), **56**, 814–819 © The Macmillan Press Ltd., 1987

### Breast cancer and atypia among young and middle-aged women: A study of 110 medicolegal autopsies

M. Nielsen[1], J.L. Thomsen[2], S. Primdahl[1], U. Dyreborg[3], & J.A. Andersen[4]

[1]*Department of Pathology, Frederiksberg Hospital, Copenhagen, DK-2000 F;* [2]*University Institute of Forensic Medicine, Copenhagen, DK-2100 Ø; and Departments of* [3]*Radiology and* [4]*Pathology, Odense University Hospital, Odense, DK-5000 C, Denmark.*

**Summary** In 110 consecutive, medicolegal autopsies of young and middle-aged women (range 20–54 years) the breasts were examined by an extensive histopathologic method and by correlative specimen radiography. Malignancy was found in 22 women (20%) of which only one was known to have had clinical invasive breast cancer (IBC). At autopsy 2 women had IBC (2%), the remaining *in situ* carcinoma (*in situ* BC) of microfocal type (18%), i.e. 15 (14%) intraductal carcinomas (DCIS), 4 (3%) lobular carcinoma *in situ* (LCIS) and one (1%) both DCIS and LCIS. Forty-five per cent of the women with malignancy had multicentric and 41% had bilateral lesions. Forty-five per cent of all histologically confirmed malignant lesions were identified by specimen radiography. Adenosis, benign epithelial hyperplasia, papilloma and duct ectasia were positively associated with malignancy. In addition malignancy was significantly more frequent among women aged more than 40 years, with late age at first full-term pregnancy, with alcohol abuse and with steatosis or cirrhosis of the liver. The results suggest that clinically occult *in situ* BC are frequent in young and middle-aged women.

The life-long cumulated frequency of clinical, invasive breast cancer (IBC) is 6.5% for Danish women (Danish Cancer Registry, 1983), whereas estimates of the frequency of noninvasive lesions, i.e. intraductal carcinoma (DCIS), lobular carcinoma *in situ* (LCIS) and atypical epithelial hyperplasia, are still insufficient. Only a few decades ago DCIS and LCIS were infrequently reported lesions but they have recently attracted much more attention. The available information stems mainly from studies using mammography for mass screening (Andersen, 1981; Moskowitz, 1981; Tabár *et al.*, 1985), and from clinically based series of self-selected women (Betsill *et al.*, 1978; Fisher *et al.*, 1986; Moskowitz, 1983; Page *et al.*, 1978; Page *et al.*, 1982). However, as *in situ* breast carcinoma (*in situ* BC) is rarely symptomatic and the sensitivity of mammography in detecting malignancy is limited despite improved techniques (Holland *et al.*, 1983), much uncertainty exists concerning these lesions.

Histopathologic examination of the breast is a reliable method for detection of clinically and radiologically occult malignant and atypical lesions but extensive sampling is mandatory to detect these small lesions. The few autopsy studies using this technique have been carried out mainly on elderly women (Alpers & Wellings, 1985; Kramer & Rush, 1973; Nielsen *et al.*, 1984).

To obtain an estimate of the frequency and characteristics of clinically occult malignant and atypical lesions in younger Danish women, a series of medicolegal cases seemed to be a suitable sample. The results of an extensive histopathologic breast tissue examination and correlative specimen radiography in such a group of women are presented in this paper.

### Materials and methods

The study group consisted of a series of 110 consecutive medicolegal autopsies on Caucasian Danish women, performed from October 1983 to July 1984 at the University Institute of Forensic Medicine in Copenhagen. The criteria for exclusion were age younger than 20 years or older than 54 years, death more than 6 days before autopsy and extensive injury to one or both breasts. The Forensic Institute in Copenhagen covers an area with a population of 2.3 million with about 605,000 women between 20 and 54 years of age (Danmarks Statistik, 1985). During the sampling period about 10% of all deaths within the area underwent medicolegal examination and of these about 25% were subjected to autopsy.

During the medicolegal autopsy, bilateral total mastectomy with partial axillary dissection (Cady, 1973) was performed in all cases, except for one woman who had undergone previous mastectomy for IBC and consequently only had a contralateral mastectomy.

Each fresh breast specimen was weighed and radiographed intact in a single frontal projection in a Faxitron (model 43805N) using Kodak industrial M film. With the nipple as the center point, the specimen was then divided into the four quadrants and cut systematically from the deep fascia to the outer surface in 5 mm-thick slices. Each of these were radiographed and thereafter fixed in formalin. The slices were examined grossly after fixation and the relative proportion of glandular and fatty breast tissue was evaluated in 10% estimates. All tissue including fatty tissue was processed routinely for paraffin embedding. Axillary lymph nodes were also radiographed and processed for microscopic examination.

The total number of paraffin blocks was 60,335 with each block containing ~1.3 g of breast tissue. The average number of paraffin blocks from each breast specimen was 275 (range 57–683). The average number of lymph nodes from each woman was 21 (range 0–50). Sections for microscopic examination were cut from each paraffin block and stained with hematoxylin and eosin. In cases with suspicion of or with manifest *in situ* BC and atypical ductal hyperplasia (ADH), additional serial sections were prepared. The PAS-alcian stain was used to support the differentiation between LCIS and DCIS, based on the presence of intracytoplasmic lumina (Andersen & Vendelboe, 1981).

The microscopic changes were evaluated according to the guidelines of World Health Organization (WHO, Sobin, 1981) and Azzopardi (1979). Various degrees of autolysis did occur, but in a large number of cases the autolytic changes, such as lighter staining of the cytoplasm and shrinkage of the nuclei, did not pose serious problems for the histologic evaluation.

The X-ray films were evaluated blindly by one of the authors (U.D.).

A lesion was denoted microfocal if the diameter was 5 mm or less. Multicentricity was defined as the occurrence of

Correspondence: M. Nielsen.
Received 18 August 1987.

separate foci of one type of lesion in more than one quadrant in one breast.

The occurrence of apocrine metaplasia, adenosis, benign and atypical epithelial hyperplasia, papilloma, duct ectasia, radial scar, mastitis, adenoma of the nipple and fibroadenoma were assessed as well.

In the one case of previous surgical mastectomy, the available histologic slides were reviewed. Clinical data and autopsy findings were obtained from police reports, death certificates, autopsy reports, hospital records and general practitioners.

Statistical analysis was carried out by chi-square, Student's *t*-test and Mann–Whitney test.

### Results

The overall frequency of malignant breast lesions among the 110 women was 20% (22 women). Another 7% (8 women) had ADH only (Table I).

At autopsy IBC was diagnosed in 2 women (2%). One of them had been treated for ductal IBC and had at the autopsy a primary ductal IBC of the contralateral breast, measuring 15 mm in diameter. The other woman had not had any symptoms of breast disease, but both diffuse and tumour-forming DCIS (Figure 1) and LCIS with foci of micro-invasion were found as multiple lesions in both breasts.

Twenty women (18%) had *in situ* BC and only one of them was recorded to have had a breast biopsy previously, showing chronic fibrocystic disease. Table I shows that 15 women had DCIS (Figures 2–5), four LCIS (Figures 6 & 7) and one both DCIS and LCIS in the same breast. The growth pattern of the individual lesion was microfocal in all cases. Predominant cribiform configuration of DCIS was noted in 4 cases (25%), solid in 2 cases (12%) and combinations of comedo, papillary, clinging, solid and cribiform configurations in 10 cases (63%). Prominent lymphoid infiltration occurred in two cases (10%) and in another one (5%) several ducts with DCIS exhibited signs of regression by a strong desmoplastic periductal reaction.

**Table I** Number of women with primary malignant and atypical breast lesions among 110 medicolegal autopsy cases.

| *Type of lesion* | *No. of women* |
|---|---|
| Invasive breast cancer (IBC) | 2 |
| Intraductal carcinoma (DCIS) | 15 |
| Lobular carcinoma *in situ* (LCIS) | 4 |
| DCIS/LCIS | 1 |
| Total number of malignant lesions | 22 (20%) |
| Atypical ductal hyperplasia (ADH) | 8 (7%) |

**Figure 1** Fifty-year-old woman. Part of tumour-forming intraductal carcinoma of cribiform and solid type, 5.5 mm in diameter (H & E, ×480).

**Figure 2** Fifty-one-year-old woman. Intraductal carcinoma of clinging, cribiform and solid type, 0.7 mm in diameter (H & E, ×480).

**Figure 3** Fifty-year-old woman. Intraductal carcinoma of clinging, cribiform and solid type, 0.6 mm in diameter (H & E, ×480).

**Figure 4** Thirty-nine-year-old woman. Cribiform intraductal carcinoma, 2.4 mm in diameter (H & E, ×480).

Of the 8 women with ADH only, one had a clinical diagnosis of breast disease, a non-biopsied lump. Of the remaining 80 women without malignant and atypical lesions 5 had a diagnosis of various benign breast diseases, confirmed by a previous biopsy in only one case.

Of the 22 women with malignancy, 10 (45%) had multicentric and 9 (41%) bilateral lesions (Table II). Except one, all women with bilaterality had multicentric lesions. In 59% the malignant lesions were located centrally and in 36% in the upper lateral quadrant. Of the 8 women with ADH only, 5 had multicentric and 3 bilateral lesions (Table II).

All women with malignancy had benign epithelial hyper-

**Figure 5** Thirty-year-old woman. Clinging and cribiform intraductal carcinoma, 0.9 mm in diameter (H & E, × 120).

**Figure 6** Fifty-year-old woman. Part of focus with lobular carcinoma *in situ*, 2.0 mm in diameter (H & E, × 480).

**Figure 7** Forty-nine-year-old woman. Part of focus with lobular carcinoma *in situ*, 1.5 mm in diameter (H & E, × 480).

plasia elsewhere in the same breast. ADH was also found in all cases of malignancy except for one with LCIS. Atypical lobular hyperplasia was not found in any case. Adenosis, benign epithelial hyperplasia, papilloma and duct ectasia were all significantly more frequent among women with malignancy (Table III).

**Table II** Distribution of malignant and atypical breast lesions among 110 medicolegal autopsy cases

| | *IBC* | *DCIS* | *LCIS* | *DCIS/LCIS* | *ADH* |
|---|---|---|---|---|---|
| No. of women | 2 | 15 | 4 | 1 | 8 |
| Bilaterality | 2 | 5 | 0 | 1 | 3 |
| Multicentricity | 2 | 7 | 0 | 1 | 5 |

[a]For abbreviations, see **Table I**

**Table III** Histologic parameters among 110 women with and without breast cancer

| | *Breast cancer* | | |
|---|---|---|---|
| | *With* (*n* = *22*) | *Without* (*n* = *88*) | *P* |
| Apocrine metaplasia | 21 (95%) | 75 (85%) | NS |
| Adenosis | 17 (77%) | 41 (47%) | <0.01 |
| Benign epithelial hyperplasia | 22 (100%) | 48 (55%) | <0.0005 |
| Atypical epithelial hyperplasia | 21 (95%) | 8 (9%) | <0.0001 |
| Papilloma | 12 (55%) | 26 (30%) | <0.05 |
| Duct ectasia | 19 (86%) | 54 (61%) | <0.05 |
| Radial scar | 7 (32%) | 24 (27%) | NS |
| Mastitis | 0 | 2 (2%) | NS |
| Adenoma of the nipple | 0 | 2 (2%) | NS |
| Fibroadenoma | 4 (18%) | 20 (23%) | NS |

None of the women in the series had axillary lymph node metastases.

In none of the cases could a palpable or visual abnormality at the macroscopic examination be ascribed to lesions diagnosed as malignant or atypical.

Changes which would have called for a breast biopsy if they had been observed *in vivo* on a mammogram, were found in 23 women (11%) by correlative specimen radiography (Table IV). In 10 of these cases malignancy was confirmed by microscopy (Pv pos = 43%). Five DCIS and one ADH were found because of microcalcifications and two DCIS and one ADH because of soft tissue densities, which proved to be fibrous areas with the occurrence of malignant or atypical ducts. One LCIS was found within an area of soft tissue density caused by benign epithelial proliferation. Of the 87 women with negative radiologic examination, 12 had microfocal *in situ* BC (Pv neg = 86%). The sensitivity was 0.45 (10/22 cases of malignancy) and the specificity 0.85 (75/88 cases without malignancy).

The median age of the whole group was 39 years at autopsy (range 20–54 years). The median age of the women with malignancy was 45 years compared to 37 years for the rest of the series, and this difference was statistically significant ($P<0.001$). The median age for women with ADH was 42 years, and this was not significantly different from women without malignancy or ADH. Table V shows the distribution of IBC, *in situ* BC and ADH stratified by age. Only 3 cases of *in situ* BC and ADH, respectively, were observed in the group aged from 20 to 39 years.

One woman in the series had a family history of IBC (mother) and had DCIS diagnosed at autopsy.

Twenty-three women (28%) were older than 25 years at first full-term pregnancy. Late age at first pregnancy was positively associated ($P<0.02$) with malignancy (9/23 women or 39%) compared to women without (9/60 women or 15%).

Parity, oral contraceptives, disorders of the reproductive system, height, overweight (≥10% deviation from the recommended standard (Natvig, 1956)), weight per breast and the relative amount of glandular and fatty breast tissue were parameters registered, which were not associated with an increased frequency of malignancy and ADH.

Alcoholics were common in the series (45%) and the frequency of alcoholism and of cases with steatosis and/or cirrhosis of the liver was significantly increased among women with malignancy ($P<0.02$ and $P<0.02$, respectively, Table VI). The median age of the women with and without alcohol abuse was comparable (40 and 38 years, respectively). We have no detailed information on the duration, amount, type (wine, beer, spirits) and pattern of the alcohol consumption. Changes of the liver were evaluated by gross inspection and in some cases by microscopic examination as well. Steatosis and/or cirrhosis were associated with a history of alcohol abuse in all cases.

**Table IV** Radiologic changes by specimen radiography among 110 women with and without breast cancer

| | *Number of women with breast cancer (n=22)* | *Number of women without breast cancer (n=88)* |
|---|---|---|
| Microcalcifications | | 7 |
| IBC[a] | 1 | |
| DCIS | 4 | |
| DCIS/LCIS | 1 | |
| Soft tissue density | | 6 |
| IBC | 1 | |
| DCIS | 2 | |
| LCIS | 1 | |
| Total number of women with suspicious changes | 10 | 13 |
| Number of women with no suspicious changes | 12 | 75 |
| DCIS | 9 | |
| LCIS | 3 | |

[a]For abbreviations, see **Table I**

**Table V** Age distribution of 110 women with and without malignant and atypical breast lesions

| *Years* | *Total number of women (n=110)* | *Number of women with breast cancer (n=22)* | *Number of women with atypical lesions (n=8)* |
|---|---|---|---|
| 20–29 | 23 | 0 | 1 |
| 30–39 | 36 | 3 (8%) | 2 |
| 40–49 | 33 | 13 (39%) | 4 |
| 50–54 | 18 | 6 (33%)[a] | 1 |

[a]Two women had invasive breast cancer

**Table VI** Number of women with alcohol abuse and steatosis and/or cirrhosis among 110 medicolegal autopsy cases with or without breast cancer

| | *Women with breast cancer (n=22)* | *Women without breast cancer (n=88)* |
|---|---|---|
| +alcohol abuse | 15 (68%) | 35 (40%) |
| −alcohol abuse | 7 (32%) | 53 (60%) |
| +steatosis/cirrhosis | 12 (55%) | 23 (26%) |
| −steatosis/cirrhosis | 10 (45%) | 65 (74%) |

Fifty-seven women (51%) had abused drugs (antidepressants, analgetics, anxiolytics and narcotics), frequently in combination with alcohol. Abuse of drugs was not associated with an increased occurrence of malignancy.

Table VII shows the modes of death. Twenty-five per cent were natural deaths, 70% unnatural deaths and for the remaining 5% unknown. No significant difference was found regarding the mode of death in women with and without malignancy.

### Discussion

This extensive histopathologic study of a series of medicolegal autopsies gives an estimate of the occurrence of clinically occult malignant and atypical breast lesions among young and middle-aged Danish women. *In situ* BC and ADH produced no grossly identifiable abnormalities, in agreement with the fact that most of the lesions were microfocal, i.e. of a diameter of 5 mm or less. For the same reason many of the lesions were also undectable by

**Table VII** Modes of death among 110 medicolegal autopsy cases with and without breast cancer

| | *Number of women without breast cancer (n=88)* | *Number of women with breast cancer (n=22)* |
|---|---|---|
| Natural death | 22 (25%) | 6 (27%) |
| cardiovascular disease | 11 | 1 |
| cerebral vascular disease | 4 | 2 |
| diabetes | 1 | 0 |
| infectious disease | 6 | 3 |
| Unnatural death | 61 (69%) | 16 (73%) |
| accident | 33 | 9 |
| suicide | 23 | 5 |
| homicide | 5 | 2 |
| Unknown | 5 (6%) | 0 |

correlative specimen radiography, as *in situ* BC and ADH frequently are outside the radiologically detectable size range. However, the proportion of radiologically false negative *in situ* BC (55%) is within the wide range reported in the literature, where 15–67% of the cases are found to be occult (Holland *et al.*, 1983).

Except for a hypothetic, alcohol induced increase in the frequency of cases of breast malignancy, we have no reason to suspect any selection of high risk women for IBC in the current study (Danielson *et al.*, 1982, Dansk lægemiddelstatistik, 1985; Hardt, pers. comm.; Kelsey, 1979; Lynge & Thygesen, pers. comm.; Medicinalstatistiske meddelelser, 1973; Mouridsen & Blichert-Toft, 1982; Nielsen *et al.*, 1986; Osler, 1986; Page *et al.*, 1978; Rosenberg *et al.*, 1982; Sattin *et al.*, 1986; Williams & Horum, 1977). The majority of the women (75%) had died outside hospital institutions and as might be expected there was a preponderance of non-natural deaths (70%). None of these facts, however, can be expected directly to cause a selection of high risk women, and a Danish study (Asnæs and Paaske, 1980) does not indicate a high frequency of unexpected malignant diseases in medicolegal deaths.

Using an extensive histopathologic technique on autopsy cases, Kramer & Rush (1973) found the frequency of occult IBC to be 1.4%, of DCIS 4.3% and of ADH 10% among elderly women. Among random autopsy cases of women, Alpers & Wellings (1985) detected no unexpected IBC, but 5.9% had DCIS. In none of these studies was LCIS diagnosed. In a previous hospital based study of elderly women, we found the life-long cumulated frequency of breast malignancy to be as high as 25% (Nielsen *et al.*, 1984). The occult malignant lesions included 1.3% IBC and 18% *in situ* BC, of which 4% were LCIS and another 4% combinations of DCIS and LCIS. The frequency of atypical lesions was 4%.

In the current study a similar but even more extensive sampling technique including fatty breast tissue was carried out, because we from previous studies were impressed by the fact that *in situ* BC were often microfocal and limited to a few small ducts and/or lobules. The frequency of clinical IBC among young and middle-aged women was similar to the incidence among Danish women in this age group (0.8%, Danish Cancer Registry, 1983). Occult IBC was infrequent and comparable to the frequency in the elderly women, whereas the frequency of clinically occult *in situ* BC was at least twice the life-long cumulated incidence of clinical IBC (6.5%, Danish Cancer Registry, 1983). The ratio between LCIS and DCIS was 1:4 and comparable to the known ratio for lobular and ductal IBC, in agreement with the results of recent surgical and screening studies (Andersen, 1981; Moskowitz, 1981; Rosen *et al.*, 1980; Schwartz *et al.*, 1980; Tabár *et al.*, 1985).

Elderly women have a much higher incidence of IBC than young and middle-aged women, but this does not necessarily imply a correspondingly higher frequency of *in situ* BC and atypical lesions as our results indicate. Stratification by age showed that women younger than 40 years in the current study did have a very low frequency of ADH and *in situ* BC only (Table V). On the other hand, middle-aged women had a high frequency compared to the figures for the elderly women (Alpers & Wellings, 1985; Kramer & Rush, 1973; Nielsen *et al.*, 1984).

The frequency of multicentricity of DCIS (50%, Table II) is in keeping with recent studies (Lagios *et al.*, 1982; Schwartz *et al.*, 1980). However, the frequency of bilaterality of DCIS (40%, Table II), was higher than in other studies (Urban, 1969; Webber, *et al.*, 1981), a fact which might be due to the more extensive tissue sampling of both breasts. Because of the small number of lesions, no meaningful conclusions can be drawn regarding LCIS.

As emphasized in previous studies (Gallager, 1980; Kramer & Rush, 1973) malignancy was also in the current study found almost exclusively in breasts with benign intraductal hyperplasia (Table III), which seems to be a prerequisite for IBC development. The high frequency of *in situ* BC and ADH, which have morphologic features in common and no strict criteria to distinguish between them in some cases, is in agreement with the hypothesis that the majority of IBC pass through a sequence of histologically recognizable changes (Gallager, 1980). The frequent localization within small ducts and/or lobules and the morphologic similarities between microfocal DCIS and LCIS fit well with the hypothesis of Welling & Jensen (1973) that both lesions arise in the lobular-terminal unit. The registered association of malignancy with papillomas confirms the results of other studies (Haagensen *et al.*, 1981; Ohuchi *et al.*, 1984), but the number of cases were too small to evaluate the effect of these lesions independent of age (Table III). The same was true for the association with adenosis and duct ectasia (Table III).

Information on subsequent development of IBC in women with biopsy treated small DCIS of the type presented in our study is currently insufficient. A few long-term follow-up studies indicate the risk to be about 25–30%, i.e. the same as for LCIS (Andersen & Ottesen, unpublished data; Andersen & Schiødt, 1980; Betsill *et al.*, 1978; Page *et al.*, 1982; Rosen *et al.*, 1980). Assuming that IBC only evolves in breasts with *in situ* BC in young and middle-aged women, our high frequency of *in situ* BC is not incompatable with such estimates. Although our results indicate that the noninvasive phase may be long, i.e. they appear 15–20 years before the median age of women with clinical IBC or be lifelong, this need not apply to *in situ* BC hypothetically formed after the menopause.

The current study provides a solid basis for the evaluation of the frequency of clinically occult malignant and atypical breast lesions. The results indicate that *in situ* BC and ADH are frequent among middle-aged women. Further studies are mandatory to get more insight into the natural history of IBC, as noninvasive lesions present a difficult therapeutic dilemma and there is no way at present to predict which lesions will progress to IBC.

Supported by grants from the Danish Medical Research Council (12-4720) and Mrs Astrid Thaysens Foundation (ATL 09/84). The authors thank Mrs Inga Snetoft for secretarial help.

### References

ALPERS, C.E. & WELLINGS, S.R. (1985). The prevalence of carcinoma *in situ* in normal and cancer-associated breasts. *Hum. Pathol.*, **16**, 796.

ANDERSEN, I. (1981). Radiologic screening for breast carcinoma. *Acta Radiol. Diag.*, **22**, 227.

ANDERSEN, J.A. & OTTESEN, G.L. (1988). Ductal carcinoma *in situ*: A lifelong follow-up of 30 biopsy treated cases (unpublished data).

ANDERSEN, J.A. & SCHIØDT, T. (1980). On the concept of carcinoma *in situ* of the breast. *Path. Res. Pract.*, **166**, 407.

ANDERSEN, J.A. & VENDELBOE, M.L. (1981). Cytoplasmic mucous globules in lobular carcinoma *in situ*. Diagnosis and prognosis. *Am. J. Surg. Pathol.*, **5**, 251.

ASNÆS, S. & PAASKE, F. (1980). A systematic autopsy study. *Forensic Sci.*, **15**, 3.

AZZOPARDI, J.G. (1979). *Problems in breast pathology*, vol. II, pp. 113 & 192. W.B. Saunders: New York.

BETSILL, W.L., ROSEN, P.P., LIEBERMAN, P.H. & ROBBINS, G.F. (1978). Intraductal carcinoma. Long-term follow-up after treatment by biopsy alone. *J. Amer. Med. Assoc.*, **239**, 1863.

CADY, B. (1973). Total mastectomy and partial axillary dissection. *Surg. Clin. North. Am.*, **53**, 313.

DANIELSON, D.A., JICK, H., HUNTER, J.R., STERGACHIS, A. & MADSEN, S. (1982). Nonestrogenic drugs and breast cancer. *Am. J. Epidemiol.*, **116**, 329.

DANISH CANCER REGISTRY (1983). *Cancer incidence in Denmark 1978, 1979 and 1980.* Danish Cancer Society: Copenhagen,

DANMARKS STATISTIK (1985). *Statistical Yearbook*. Copenhagen, vol. **89**.

DANSK LÆGEMIDDELSTATISTIK (1985). *Lægemiddelforbruget* i Danmark, p. 70.

FISHER, E.R., SASS, R., FISHER, B., WICKERHAM, L., PAIK, S.M. & COLLABORATING NSABP INVESTIGATORS (1986). Pathologic findings from the National Surgical Adjuvant Breast Project (protocol 6) I. Intraductal carcinoma (DCIS). *Cancer*, **57**, 197.

GALLAGER, H.S. (1980). The developmental pathology of breast cancer. *Cancer*, **46**, 905.

HAAGENSEN, C.D., BODIAN, C. & HAAGENSEN, D.E. (1981). Breast carcinoma. Risk and detection, p. 197. W.B. Saunders: London.

HOLLAND, R., HENDRICKS, J.H.C. & MRAVUNAC, M. (1983). Mammographically occult breast cancer. A pathologic and radiologic study. *Cancer*, **52**, 1810.

KELSEY, J.L. (1979). A review of the epidemiology of human breast cancer. *Epidemiol. Rev.*, **1**, 74.

KRAMER, W.M. & RUSH, B.F. (1973). Mammary duct proliferation in the elderly. A histopathologic study. *Cancer*, **31**, 130.

LAGIOS, M.D., WESTDAHL, P.R., MARGOLIN, F.R. & ROSE, M.R. (1982). Duct carcinoma *in situ*. Relationship of extent of noninvasive disease to the frequency of occult invasion, multicentricity, lymph node metastases, and short-term treatment failures. *Cancer*, **50**, 1309.

MEDICINALSTATISTISKE MEDDELELSER (1973). 1, Sundhedsstyrelsen, Denmark: *Medicinsk fødselsstatistik 1970*, p. 28.

MOSKOWITZ, M. (1981). Mammographic screening: Significance of minimal breast cancers. *Am. J. Roentgenol.*, **136**, 735.

MOSKOWITZ, M. (1983). The predictive value of certain mammographic signs in screening for breast cancer. *Cancer*, **51**, 1007.

MOURIDSEN, H.T. & BLICHERT-TOFT, M. (1982). *Brystkræft. Diagnostik og behandling*, p. 17. Roche: Copenhagen.

NATVIG, N. (1956). *Nye høyde-vekttabeller for norske kvinner og menn.* Landsforeningen for kosthold og helse. Oslo.

NIELSEN, M., JENSEN, J. & ANDERSEN, J. (1984). Precanceraous and cancerous breast lesions during lifetime and at autopsy. *Cancer*, **54**, 612.

NIELSEN, M., CHRISTENSEN, L. & ANDERSEN, J. (1986). Contralateral cancerous breast lesions in women with clinical invasive breast carcinoma. *Cancer*, **57**, 897.

OHUCHI, N., ABE, R., & KASAI, M. (1984). Possible cancerous change of intraductal papillomas of the breast. A 3-D reconstruction study of 25 cases. *Cancer*, **54**, 605.

OSLER, M. (1986). Overvægt – et dansk sundhedsproblem. *Ernæringsnyt*, **48**, 2.

PAGE, D.L., DUPONT, W.D., ROGERS, L.W. & LANDENBERGER, M. (1982). Intraductal carcinoma of the breast: Follow-up after biopsy only. *Cancer*, **49**, 751.

PAGE, D.L., ZWAAG, R.V., ROGERS, L.W. & 3 others (1978). Relation between component parts of fibrocystic disease complex and breast cancer. *J. Natl Cancer Inst.*, **61**, 1055.

ROSEN, P.P., BRAUN, D.W. JR. & KINNE, D.E. (1980). The clinical significance of preinvasive breast carcinoma. *Cancer*, **46**, 919.

ROSENBERG, L., SLONE, D., SHAPIRO, S. & 8 others (1982). Breast cancer and alcoholic-beverage consumption. *Lancet*, **i**, 267.

SATTIN, R.W., RUBIN, G.L., WINGO, P.A., WEBSTER, L.A. & ORY, H.W. (1986). Oral-contraceptive use and the risk of breast cancer. The cancer and steroid hormone study of the centers for disease control and the National Institute of Child Health and Human Development. *N. Engl. J. Med.*, **315**, 405.

SCHWARTZ, G.F., PATCHESFSKY, A.S., FEIG, S.A., SHABER, G.S. & SCHWARTZ, A.B. (1980). Multicentricity of non-palpable breast cancer. *Cancer*, **45**, 2913.

SOBIN, L.H. (1981). *Histological Typing of Breast Tumours*, 2nd ed. WHO: Geneva.

TABÁR, L., FAGERBERG, G.J.G., GAD, A. & 9 others (1985). Reduction in mortality from breast cancer after mass screening with mammography. *Lancet*, **i**, 829.

URBAN, J.A. (1969). Bilateral breast cancer. *Cancer*, **24**, 1310.

WEBBER, B.L., HEISE, H., NEIFELD, J.P. & CASTA, J. (1981). Risk of subsequent contralateral breast carcinoma in a population of patients with *in-situ* breast carcinoma, *Cancer*, **47**, 2928.

WELLINGS, S.R. & JENSEN, H.M. (1973). On the origin and progression of ductal carcinoma in the human breast. *J. Natl Cancer Inst.*, **50**, 1111.

WILLIAMS, R.G. & HORUM, J.W. (1977). Association of cancer sites with tobacco and alcohol consumption and socioeconomic status of patients: Interview study from the Third National Cancer Survey. *J. Natl. Cancer Inst.*, **58**, 527.